# When the Gut Took a Wrong Turn to the Right: A Case Report of a Rare Complication of Closed Loop Small Bowel Obstruction With Small Bowel Volvulus in a Right Paraduodenal Hernia With Midgut Malrotation

**DOI:** 10.7759/cureus.53342

**Published:** 2024-01-31

**Authors:** Samanvitha H, Sushmitha Puttappa Shivagange, Shantkumar S Sajjan, Naveen S G, Harsha M T

**Affiliations:** 1 Department of Diagnostic Radiology, Bangalore Medical College and Research Institute, Bengaluru, IND; 2 Department of Diagnostic Radiology, People Tree Hospital, Bengaluru, IND; 3 Department of Diagnostic Radiology, Aster CMI Hospital, Bengaluru, IND; 4 Department of Diagnostic Radiology, Bangalore Medical College and Research Institute, Bangalore, IND; 5 Department of General Surgery, Bangalore Medical College and Research Institute, Bengaluru, IND; 6 Department of Interventional Radiology, AIl India Institute of Medical Sciences Rishikesh, Rishikesh, IND; 7 Department of Radiology, Postgraduate Institute of Medical Education and Research, Chandigarh, IND

**Keywords:** gut malrotation, volvulus, closed-loop obstruction, strangulation, small bowel obstruction, internal hernia, right paraduodenal hernia

## Abstract

Internal hernias constitute 5.8% of all small bowel obstructions. The right paraduodenal hernia is a less common subtype of the paraduodenal hernia. Lack of specific signs and symptoms precludes its clinical diagnosis, which emphasizes the need for computed tomography in diagnosis. We present a case of a 24-year-old male patient with a right paraduodenal hernia and midgut malrotation causing closed loop small bowel obstruction and small bowel volvulus within the hernial sac who underwent laparoscopy-assisted reduction of hernia and adhesiolysis with closure of the peritoneal defect. Since the right paraduodenal hernia is associated with gut malrotation, risk of strangulation, closed-loop obstruction, and rarely volvulus, these patients need prompt radiological diagnosis and surgical intervention.

## Introduction

Even though the incidence of internal hernias is less than 1%, they constitute 5.8% of all small bowel obstructions, and untreated cases with strangulation show an overall mortality of over 50% [1,2]. Right paraduodenal hernia is a less common subtype of the paraduodenal hernia and occurs commonly in the setting of nonrotated small bowel [3,4].

It is more commonly seen in males in the fourth to sixth decade with the earliest documented presentation seen in a one-week-old neonate [5,6]. Clinical presentation can be asymptomatic or with recurrent post-prandial abdominal pain or features of acute intestinal obstruction. A lack of specific signs and symptoms precludes the clinical diagnosis of a right paraduodenal hernia. Computed tomography with oral and intravenous contrast is helpful. Due to the risk of strangulation, the right paraduodenal hernia needs prompt surgical intervention.

As per our best possible research, there is no documentation of volvulus occurring within the right paraduodenal hernial sac in the literature. Shinohara et al. have documented a very rare case of a right paraduodenal hernia presenting as volvulus of the non-herniated small bowel [7]. Very few cases of volvulus have been reported in the left paraduodenal hernia.

We present a rare case of right paraduodenal hernia with midgut malrotation presenting with a rare complication of closed loop small bowel obstruction (SBO) and small bowel volvulus within the hernial sac.

## Case presentation

A 24-year-old male patient presented with vomiting and pain in the right hypochondrium and right lumbar region. He was passing stools. He had a similar episode two weeks back, which subsided on conservative management. He had no co-morbidities or previous abdominal surgeries. On examination, vitals were stable. A per abdomen examination revealed fullness in the right hypochondrium and right lumbar quadrants along with tenderness. Bowel sounds were present. Per rectal examination was normal. Other laboratory tests were normal.

**Figure 1:**
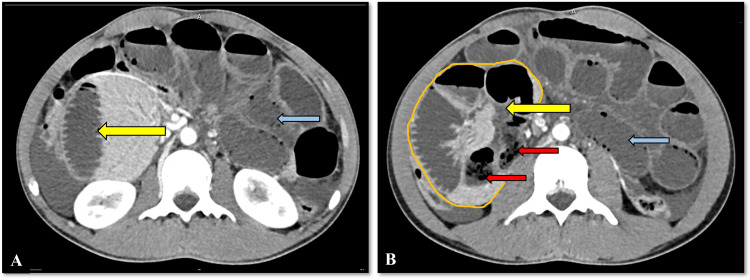
Right paraduodenal hernia Axial sections of the CECT abdomen (A, B) show a cluster of dilated distal duodenal and proximal jejunal loops on the right side of the abdomen (yellow arrows). B: A few small bowel loops in the hernial sac show a small bowel feces sign (red arrows). Also note the dilated small bowel loops in the rest of the abdominal cavity (blue arrows). CECT: contrast-enhanced computed tomography

An erect abdominal radiograph showed a few dilated small bowel loops with multiple air-fluid levels. Contrast-enhanced computed tomography (CECT) of the abdomen and pelvis was done in the late arterial and portal venous phases after administration of intravenous contrast, which showed a pseudo-encapsulated cluster of dilated distal duodenal and proximal jejunal loops in the right hypochondrium and right lumbar region (Figure 1).

**Figure 2:**
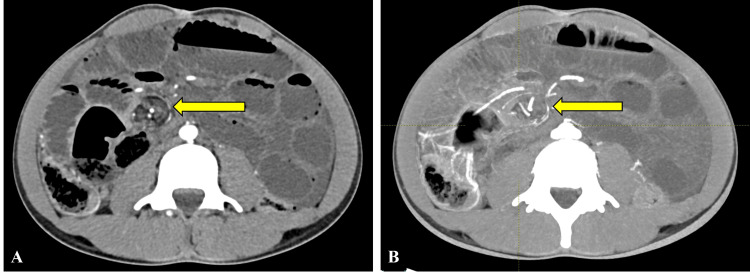
Whirlpool sign Axial sections of the CECT abdomen in thin section (A) and maximum intensity projection (B) show a swirl of mesenteric vessels (yellow arrow) near the fossa of Waldeyer, indicative of the whirlpool sign. CECT: contrast-enhanced computed tomography

**Figure 3:**
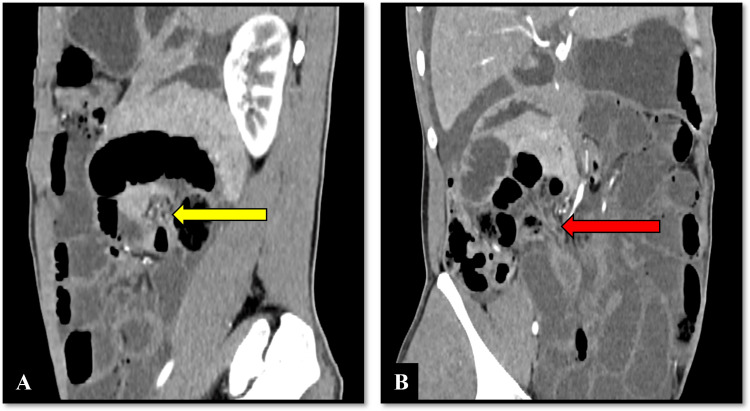
Whirlpool sign and small bowel volvulus within the right paraduodenal hernial sac Parasagittal (A) and curved planar reconstruction (B) sections of the CECT abdomen show a swirl of mesenteric vessels (yellow arrow) noted near the fossa of Waldeyer, indicating volvulus. B: Small bowel loops are noted entering and exiting the hernial sac (red arrow) causing closed-loop obstruction with volvulus. CECT: contrast-enhanced computed tomography

A swirl of mesenteric vessels within a background of mesenteric fat attenuation, indicative of a whirlpool sign (Figure 2) was seen near the fossa of Waldeyer resulting in closed bowel obstruction and small bowel volvulus within the hernial sac (Figure 3).

Small bowel loops outside the hernial sac were also dilated with multiple air-fluid levels. The bowel wall showed normal contrast enhancement without any intramural air.

**Figure 4:**
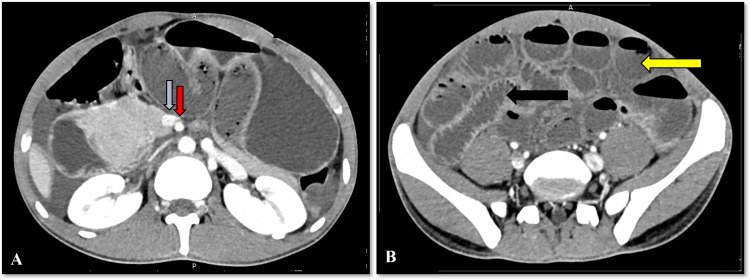
Axis of superior mesenteric vessels and gut malrotation Axial sections of the CECT abdomen show the maintained axis of the superior mesenteric artery (red arrow) and superior mesenteric vein (blue arrow). Jejunal loops are seen on the right side of the peritoneal cavity (black arrow) and ileal loops (yellow arrow) are seen on the left side of the peritoneal cavity. CECT: contrast-enhanced computed tomography

The third part of the duodenum was not visualized in the retro-mesenteric location. The duodenojejunal junction was seen to the right of the midline. Jejunal loops were predominantly on the right side and ileal loops were on the left side of the abdominal cavity with the normal position of caecum (Figure 4).

The axis of superior mesenteric vessels was maintained (Figure 4). Transverse colon and right colic vessels were seen anterior to the hernia sac with mild ascites. A radiological diagnosis of a right paraduodenal hernia with midgut malrotation causing closed-loop small bowel obstruction and small bowel volvulus within the hernial sac was made.

**Figure 5:**
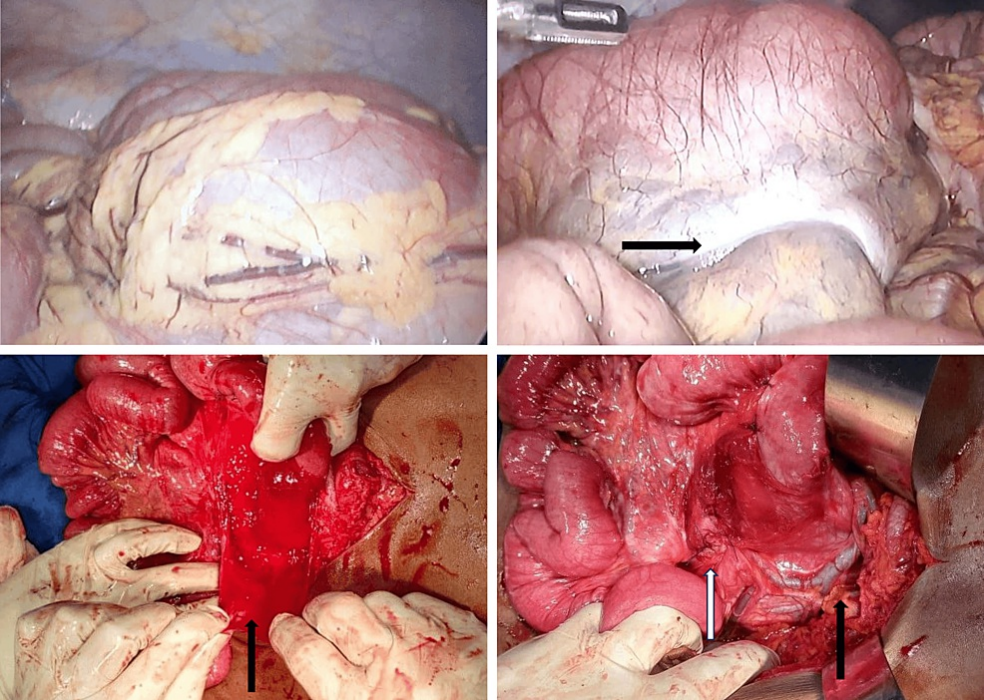
Intraoperative images A shows a glistening hernial sac containing the small bowel; B shows the neck of the hernial sac (black arrow). C shows the hernial sac (black arrow). D shows sac closure (white arrow) with superior mesenteric vessels (black arrow) seen posterior to the defect.

The patient was taken for diagnostic laparoscopy, the intraoperative findings were consistent with imaging findings. Adhesions were present between the neck of the sac and herniating bowel loops. Superior mesenteric vessels were identified posterior to the defect (Figure 5).

Laparoscopy-assisted reduction of hernia and adhesiolysis was followed by excision of the sac and closure of the peritoneal defect. As the bowel loops were viable, resection was not done. The postoperative course was uneventful.

## Discussion

Meyers et al. defined internal hernias as protrusion of viscera through an opening within the confines of the peritoneal cavity [8]. These orifices can be congenital or acquired. The most common internal hernia is paraduodenal hernia (~53% of all internal hernias) [2]. A right paraduodenal hernia is a less common subtype, constituting only 30% of the paraduodenal hernias and ~13% of all internal hernias [1,9]. Right paraduodenal hernias occur when the bowel herniates through Waldeyer’s fossa (a defect in the first part of the jejunal mesentery), behind the superior mesenteric artery and inferior to the transverse or third portion of the duodenum. Right paraduodenal hernias are usually larger and are more often fixed [8]. Clinically, both paraduodenal hernias present with chronic post-prandial pain.

During the sixth to tenth week of embryonic life, as the physiologically herniated midgut returns into the abdominal cavity, it rotates 270° in a counterclockwise direction, which finally places the pre-arterial segment (which later forms distal duodenum, jejunum and proximal ileum) in the left upper quadrant and post-arterial segment (distal ileum, cecum, ascending colon, and proximal transverse colon) in the right lower quadrant. Incomplete rotation of the pre-arterial segment, which continues to remain in the right side of the abdominal cavity, and failure of fusion between the mesentery and the third part of the duodenum, results in the fossa of Waldeyer [5,10].

Multidetector CT with intravenous contrast agent is the diagnostic imaging modality that shows an encapsulated cluster of small bowel loops in the right side of the abdominal cavity with superior mesenteric artery, middle colic artery, and ileocolic artery on the sac's anterior surface [5]. However, in our case, the defect was seen anterior to the superior mesenteric vessels, making it an atypical presentation of the right paraduodenal hernia. Clues to midgut malrotation are the absence of the normal third part of the duodenum and a leftward and ventrally placed superior mesenteric vein [11]. The cecum remains in the normal position.

C- or U-shaped configuration of bowel with beak-and-whirl sign point towards volvulus [12]. A patient is 25.3 times more likely to require surgery for SBO if they have the whirlpool sign, which denotes bowel rotation around the mesentery [13]. Due to the risk of strangulation, patients with RPH need surgical intervention. Both open and laparoscopic approaches have been described [14].

## Conclusions

Though a rare cause of intestinal obstruction, the right paraduodenal hernia is associated with gut malrotation and risk of strangulation. Other rare complications include closed-loop small bowel obstruction and volvulus. Since clinical features alone do not aid in the diagnosis, a high index of suspicion with prompt radiological diagnosis and surgical intervention is essential, even in incidentally detected patients to prevent serious complications in the future.
